# Work Rehabilitation and Medical Retirement for Myalgic Encephalomyelitis/Chronic Fatigue Syndrome Patients. A Review and Appraisal of Diagnostic Strategies

**DOI:** 10.3390/diagnostics9040124

**Published:** 2019-09-20

**Authors:** Mark Vink, Friso Vink-Niese

**Affiliations:** 1 Family and Insurance Physician, 1096 HZ Amsterdam, The Netherlands; 2 Independent Researcher, 49032 Osnabrück, Germany; frisovinkniese@googlemail.com

**Keywords:** CFS (Chronic Fatigue Syndrome), ME (Myalgic Encephalomyelitis), medical retirement, prognosis, work rehabilitation

## Abstract

Myalgic Encephalomyelitis/Chronic Fatigue Syndrome leads to severe functional impairment and work disability in a considerable number of patients. The majority of patients who manage to continue or return to work, work part-time instead of full time in a physically less demanding job. The prognosis in terms of returning to work is poor if patients have been on long-term sick leave for more than two to three years. Being older and more ill when falling ill are associated with a worse employment outcome. Cognitive behavioural therapy and graded exercise therapy do not restore the ability to work. Consequently, many patients will eventually be medically retired depending on the requirements of the retirement policy, the progress that has been made since they have fallen ill in combination with the severity of their impairments compared to the sort of work they do or are offered to do. However, there is one thing that occupational health physicians and other doctors can do to try and prevent chronic and severe incapacity in the absence of effective treatments. Patients who are given a period of enforced rest from the onset, have the best prognosis. Moreover, those who work or go back to work should not be forced to do more than they can to try and prevent relapses, long-term sick leave and medical retirement.

## 1. Introduction

Myalgic encephalomyelitis (ME) or chronic fatigue syndrome (CFS), often called ME/CFS, is a debilitating disease characterised by post-exertional malaise (PEM) with abnormally prolonged recovery after previously trivial and well tolerated exercise and activities, which differentiates ME/CFS from other fatiguing conditions [1]. Patients experience a substantial loss in quality of life, with severe disruption to occupational, social, and personal activities. It affects more women than men and in the Netherlands it is more common than multiple sclerosis (MS) [2]. There is no diagnostic test, and treatment is based on symptom management. Symptoms occurring in more than 80% of cases are muscle weakness, generalized chronic pain, cognitive dysfunction—for example concentration or short-term memory impairments, difficulty with reading or information processing—hypersensitivity to noise and/or light, new onset headaches or migraines, joint pains, dizziness and episodes of postural orthostatic hypotension [3]. The number of ME literate medical doctors is limited due to the lack of teaching about this disease in medical school and post-graduate training [4]. Most doctors are also not aware of the fact that ME has been classified as a neurological disease by the World Health Organisation [5] since 1969 with CFS as an equivalent. Many still do not believe in the disease. This is partly due to the name, chronic fatigue syndrome, which to many people suggests that patients are just a bit tired. Not many doctors will believe that a disease is serious and very disabling if it can be treated successfully by talking (cognitive behavioural therapy or CBT) and exercise (graded exercise therapy or GET). On top of this, many still think that “most people with CFS recover gradually over a period of 1–3 years” [6] or that there is a “50 to 80 percent recovery after 7 years in protracted chronic fatigue syndrome, when using the Fukuda 1994 diagnostic criteria for patient selection”, as stated in the draft report by the Australian Advisory Committee for the Chief Executive Officer of the National Health and Medical Research Council from 2018 [7]. These misconceptions can lead to all sorts of problems for patients including the refusal of benefits or medical retirement.

ME/CFS is not a rare disease [8] and it represents a considerable public health burden with an estimated annual total value of direct and indirect economic costs to society in the US of $17 to $24 billion, including $9.1 billion attributed to lost productivity [9].

The aim of this paper is to answer the following questions by reviewing the current evidence:

1. What is the prognosis of ME/CFS?
2. How does ME/CFS affect a person’s ability to work?
3. What can be expected in terms of recovery and return to work?
4. Do CBT and/or GET restore the ability to work in ME/CFS as an influential systematic review by Cairns and Hotopf from 2005 [10] advised to postpone medical retirement until patients had had a course of CBT and GET. Since then, many trials of CBT and/or GET have been published, which will enable us to answer this question.

The answers to these questions are of importance to occupational health physicians, insurance physicians, disability benefit assessors and others who evaluate adults affected by ME/CFS. As such this paper will concentrate on ME/CFS in adults.

A comprehensive search of the literature was undertaken using electronic databases (PubMed, Medline and the Cochrane Database of Clinical Trials and Web of Science) for articles on the natural history of ME/CFS, on work and occupational health in ME/CFS and on the effectiveness of CBT and GET in relation to work in studies that have been published before April 2019. We also searched the reference lists of the articles identified for the review.

## 2. Overview of ME/CFS

Myalgic Encephalomyelitis got its name after an outbreak in the Royal Free Hospital in London in 1955. The first described outbreak however happened in 1934 when 198 members of the medical and nursing staff of Los Angeles County General Hospital fell ill. The disease was initially known as atypical poliomyelitis. A prominent symptom was muscle fatigue on walking short distances and with the least exertion. The follow up of the Los Angeles cases revealed chronic disability [11].

Over the years, there have been 50 to 60 documented outbreaks, however, lately ME/CFS is mostly sporadic with occasional outbreaks [12]. It usually follows or is triggered by a viral infection, has an unknown aetiology and the onset can be acute or gradual. There are no laboratory diagnostic tests and case definitions (diagnostic criteria) are therefore used to define and diagnose ME/CFS. A group of mainly British psychiatrists came up with the Oxford criteria in 1991 [13], which are primarily used in the UK. Its only requirement is six months or more of unexplained disabling fatigue. The main characteristic of ME/CFS, postexertional malaise [1], however is not required for diagnosis. Consequently, 85% of Oxford-defined cases are healthy subjects with mild fatigue or chronic idiopathic fatigue who are misclassified as ME/CFS according to a large study by Baraniuk [14]. Both the American National Institute of Health (NIH) and the Agency for Healthcare Research and Quality (AHRQ) concluded that the Oxford criteria are flawed and that using the Oxford case definition results in a high risk of including patients who may have an alternate fatiguing illness or whose illness resolves spontaneously with time. Both agencies recommend that the Oxford definition should be retired [15,16,17].

The most commonly used diagnostic criteria are the Centers for Disease Control and Prevention (CDC) 1994 criteria, better known as the Fukuda criteria [18]. These criteria require 6 months or more of unexplained chronic fatigue and a minimum of 4 out of a list of 8 symptoms as can be seen in Table 1. However, PEM (postexertional malaise) the core symptom of ME/CFS, is only optional and not compulsory for diagnosis, as it is one of the eight additional criteria. Approximately 15% of people labelled by these criteria as having ME/CFS, were in fact healthy people [19]. Newer more restrictive criteria such as the Canadian Consensus Criteria (CCC) [12] and the International Consensus Criteria (ICC) [20] have been created which both require PEM for diagnosis, as can be seen in Table 1. The CCC and ICC select a smaller group of patients than the Fukuda criteria, and those diagnosed with ME are more impaired and less likely to suffer from depression instead of ME/CFS [21]. 

### 2.1. Advances in Understanding the Pathophysiology of ME/CFS 

For a long time, many doctors have thought that there is nothing wrong in ME/CFS because routine testing does not reveal any abnormalities. However, over the past 35 years, thousands of studies using more advanced tests have documented underlying biological abnormalities involving many organ systems in patients with ME/CFS, as noted by Komaroff in a recent overview [22]. These abnormalities include metabolic changes, immunological abnormalities in lymphocytes—especially in T cells and poorly functioning natural killer cells—and significant elevation of many blood cytokines especially in the first three years of illness which are correlated with the severity of the illness. These studies have also shown widespread neuroinflammation of the brain and cognitive impairments not explained by concomitant psychiatric disorders. Multiple studies demonstrate that during exercise the tissues of patients with ME/CFS have difficulty extracting oxygen leading to impairment of cellular energy production. This impairment is much more prominent during a second exercise tests repeated 24 h after the first [22]. Due to all these abnormalities, the American National Academy of Medicine (NAM), formerly called the Institute of Medicine (IoM), concluded in 2015 that ME/CFS is a chronic and disabling multisystem disease and not a psychiatric or psychosomatic one [23]. The Dutch Health Council came to the same conclusion in 2018 [24].

### 2.2. Misdiagnosis and under Diagnosing

The lack of a diagnostic test, the lack of standardization of the selection criteria, the lack of teaching about ME/CFS in medical school and the use of the Oxford criteria have resulted in ME/CFS becoming an umbrella term [21]. Consequently, patients with fatigue due to a psychiatric disorder, patients who experience general chronic fatigue but do not meet the other criteria, or experience fatigue as a result of an underlying medical condition, can be misdiagnosed with ME/CFS. This has profound implications, since a false positive diagnosis of ME/CFS may lead to improper interventions, withholding of treatment and a prognosis for a disease they do not have [25,26]. It also leads to the wrong impression about this disease.

It was rare for patients to get an alternative diagnosis in the clinical trials analysed by a systematic review from 2005 [10]. Since then, a number of studies have been published that specifically looked at the subject of misdiagnosis. Nacul et al. found that 24% of GP diagnosed cases did not have ME/CFS [27]. Two studies that analysed GP referrals to tertiary care showed that in 40% [25] and 49% [26] the diagnosis of ME/CFS was incorrect. Johnston et al. [28] found that in a group of 535 Australian patients diagnosed with CFS or ME by a primary care physician, 30.3% met the Fukuda criteria and only a further 32% met both the Fukuda and the ICC. In a tertiary care study by Mariman et al. [29], 228 patients who fulfilled the Fukuda criteria were assessed in a multidisciplinary integrated diagnostic pathway. Subsequently, 35.8% were diagnosed with another illness.

A number of primary-care studies showed the following, 22% of individuals, who believed they had ME/CFS, did not comply with either the Fukuda or Canadian Consensus Criteria [30]. Only 30% of patients who presented to their general practitioners (GPs) with six months or more of unexplained fatigue, had Fukuda defined ME/CFS [31]. But it is not only GPs who get the diagnosis wrong. 21% diagnosed with ME/CFS by one of four specialist physicians in tertiary care got alternative medical (2%) and psychiatric (19%) diagnoses [32].

A number of follow-up studies also reported misdiagnosis. For example, this was 10% in a nine-year follow-up study [33], 23.1% in a three-year follow-up study [34] and 24.5% in a five-year follow-up study [35]. Common alternative medical diagnoses are fatigue associated with a chronic disease, obstructive sleep apnoea, depression or anxiety [25,26,29,34]. These high rates of misdiagnosis underline the importance of evaluating differential diagnoses [35] especially when patients present with new or worsening symptoms. 

At the same time, assigning a diagnosis of ME/CFS in the current clinical setting often takes years, as there is no diagnostic test and many physicians are uninformed or misinformed about the disease. Consequently, an estimated 84–91% of patients affected by ME/CFS are not diagnosed with the disease [9].

### 2.3. Predictors of Outcome

A range of predictors of good and poor outcome have been identified and grouped into a few broad categories [10].

#### 2.3.1. Illness Management in the Initial Stages

The most important prognostic factor is how the illness is managed in its initial stages according to Dr. Ramsay [36], the infectious disease specialist involved in the management of the almost 300 patients, mainly doctors and nurses, who fell ill during the outbreak in the Royal Free Hospital in London in 1955. He also noted that most patients will try to go back to work in the initial stages when they are improving. With many other illnesses that does not pose a problem, yet with ME/CFS it does. Patients who have a period of enforced rest in the initial stages of their illness tend to have the best prognosis [36].

#### 2.3.2. Demographics

Older age was predictive of a worse outcome in a number of studies [37,38,39,40,41] but other studies reported that there was no association between age and outcome [32,42,43,44]. However, analysis of the outcome of treatments in the National Health Service (NHS) CFS clinics (*n* = 1643) by Crawley et al. [45] revealed that older age, increased pain and physical function at assessment were associated with poorer physical function at follow-up. Analysis of the data from the UK CFS/ME National Outcomes Database (*n* = 2170) in 2011 by Collin et al. [46] showed that men and people in older age groups were more likely to have ceased employment due to their fatigue-related symptoms.

#### 2.3.3. Illness Duration 

Five studies suggested that illness duration was predictive of a worse outcome [32,38,42,47,48] but this finding was not supported by five other studies that reported no association [34,49,50,51,52]. However, the large aforementioned study by Collin et al. [46] from 2011 found that illness duration was predictive of a worse outcome.

#### 2.3.4. Psychiatric Comorbidity

Having a comorbid psychiatric disorder at baseline is associated with a poorer outcome according to a systematic review by Cairns and Hotopf [10].

#### 2.3.5. Illness Severity

Approximately, 25% of ME/CFS patients are severely affected and are homebound or bedbound and dependent on others [53]. Severity is a major factor affecting prognosis [54]. In general, markers of a more severe illness (chronic symptoms, severe disability, more severe fatigue and more physical symptoms) tend to be associated with a poor outcome [30,41,44,47,48,55]. This was confirmed by the above-mentioned evaluation of treatments in the NHS CFS clinics (*n* = 1643) by Crawley et al. [45]. Leone et al. [56] found that physical functioning at baseline, deterioration of physical functioning between the baseline measurement and 12-month follow-up predicted work disability at 4-year follow-up. Hill et al. [47] who studied the natural history of severe ME, concluded that the prognosis for recovery was extremely poor. 

## 4. ME/CFS and the Occupational Health Physician

### 4.1. Sickness Absence

Occupational health physicians might have to advise on issues such as sickness absence, fitness for work, work rehabilitation and medical retirement in patients who present with chronic fatigue (CF). Most of them do not suffer from ME/CFS. Postexertional malaise, the main characteristic of ME/CFS, is the single most important factor in discriminating ME/CFS from idiopathic CF or psychiatrically explained CF. Moreover, it is also an important prognostic indicator of poorer outcome at follow-up [57].

From an occupational health point of view, it is important to know that ME/CFS can differ from client to client but also that impairments can fluctuate in nature and severity throughout its course. Symptoms can be such that they can make it difficult for clients to participate in assessments that involve effort and concentration. For this reason, assessments should usually be brief, straightforward and require minimal effort. There may be a need to break longer assessments into smaller segments of 10–20 minutes. For many ME/CFS patients it is difficult to travel. Thus, in-home or phone-based consultations may be viable alternatives [58].

Knowledge of prognostic factors—discussed earlier in this paper—related to occupational outcomes is important because ME/CFS often leads to absenteeism and full work incapacity [56,59]. Since the role of the occupational physicians is to advise on questions relating to work it is important to have some insight into the work-related functions that ME/CFS can affect. It is also important to realise that patients might be worried that they will be unable to perform to an acceptable standard due to the limitations imposed upon them by ME/CFS. At the same time, they might fear that work may have an adverse effect on symptoms and might cause relapses. This might not only interfere with their current capabilities but also with the prospects of eventual improvements, recovery and a return to work. A carefully planned and supervised programme of workplace rehabilitation should therefore also address these fears and problems [60,61,62]. Such a plan is also important when patients have just fallen ill, because the most important prognostic factor is how the illness is managed in its initial stages as noted before. Patients who have a period of enforced rest in the initial stages of their illness tend to have the best prognosis [36]. 

ME/CFS can interfere with work-related physical functions like walking, standing, sitting, lifting, pushing, pulling, reaching, carrying, and handling. It can also interfere with mental functions including the ability to understand, remember and carry out simple instructions, the ability to use appropriate judgment, and the ability to respond appropriately to supervision, co-workers, and usual work situations, including changes in a routine work setting [58].

### 4.2. Employment Status in ME/CFS

A large number of studies into the natural history of ME/CFS—most of them used the Fukuda criteria—also recorded employment status as can be seen in Table 2. However, most of these studies were not set up to for occupational health purposes. Consequently, many studies did not provide employment data at baseline or follow-up which led to heterogeneity in the data in Table 2. Also, a number of studies were fatigue studies containing a proportion of ME/CFS patients. An example of this is the study by Assefi et al. [63]. 37.3% (207/555) of the responders had ME/CFS. There was no follow-up, 61% worked and almost half of them worked less hours. 29% lost their job due to the illness and 30% were in receipt of illness benefits.

ME/CFS studies that did report on employment data at baseline and follow-up showed the following. Employment status did not change in a study with 42 months follow-up [40]. In two studies with 18 months and three-year follow-up respectively, employment status decreased from 31% to 24% [49] and from 63% to 55% [34]. In a study with a follow-up of 3.8 years, 36.5% (19/52) of CFS like cases returned to work [56]. In a nine-year follow-up by Anderson et al. [33], as a group, patients had not improved. 

Many studies contained a limited number of patients. However, the following two tertiary care studies contained a large number of patients. In a review by Castro-Marrero et al. [3] (*n* = 1757), 26% were employed and 63% were unable to work due to ME/CFS. In the aforementioned study by Collin et al. [46] of the NHS database (*n* = 2170), this was 41% and 50%, respectively.

Finally, studies which were done by ME Associations from Norway, The Netherlands, Australia, Britain and America [98,99,100,101,102], are described in Table 3. They confirm the findings from the long-term follow-up studies that many patients are unable to work due to their illness. Of particular interest is the study by the Dutch ME Association (*n* = 629) [100], which found that the percentage of patients who were able to work more than 40 h per week decreased from 14.8% to 0.8%. The percentage able to work 24 to 40 h decreased from 43% to 4% and the percentage of patients who were able to work 0 to 8 h increased from 1.4% to 28%. These are similar to the findings by TNO (The Netherlands Organisation for Applied Scientific Research) [2], a renowned independent Dutch research institute. In their evaluation study of 924 patients, 7% had never been on long-term sick leave. However, 2/3 of the 7% had to reduce their hours and 1/3 of them had to change their work due to ME/CFS. 23% who had been on long-term sick leave had gone back to work so that a total of 30% of patients were working. Approximately 77% of these 23%, however, needed adjustments to work. Many had to reduce their hours, were now doing sedentary and less physical work, often involving work behind a computer. Also, less people were able to work in management positions.

A study by Vercoulen et al. (1997) [95] that used the Oxford criteria, found similar employment rates for CFS (27%) as for MS (28%). It also found that 43% of CFS patients were on invalidity benefits/sick leave compared to 32% of MS patients. However, a study by Natelson et al. [80] used the much stricter 1988 CDC criteria [105], when they compared ME/CFS with MS, major depression and healthy controls. The percentages of patients that were unable to work due to illness were the following: 56% (ME/CFS), 5% (MS), 18% (depression) and 0% (healthy controls). A study by Sharpe et al. [43] showed that despite using the Oxford criteria, 65% were functionally impaired. At one-year follow-up it was found that most patients had been unable to work for prolonged periods. They were also unable to walk 90 m and 38% had abandoned employment due to their illness altogether. 

A number of studies also reported on improvements over time. In a three-year follow-up study by Nisenbaum et al. [34], 57% had a relapsing remitting course. A large study by Stoothoff et al. (*n* = 541) [89] found that 17.3% worked full or part-time; 15.9% were constantly getting worse yet 14% of those constantly getting worse, were still working; 8.5% were relapsing and remitting while only 1.9% were constantly getting better. 59.7% had a fluctuating course which is similar to the 57% found by Nisenbaum et al. [34]. A study by Clarke et al. [37] with a 2.5 year follow-up, found that 3% recovered, 38% improved and 59% got significantly worse or there was no change.

### 4.3. Work Rehabilitation

Work rehabilitation will usually need to start with a workload and number of hours of work that are dramatically reduced [60] using an individualised return to work plan taking the symptoms and specifics of the disease and the way it is affecting the individual employee into account. In particular, care should be taken to match the proposed duties in employment to the subject’s capabilities. Strenuous physical work, long working hours, rapidly changing shift patterns, work requiring sustained high levels of attention and concentration are likely to place sustained high pressure on the employee and are inadvisable or at least require careful monitoring until it is clear that the employee is able to sustain this level of work. Care must be taken not to set definite deadlines in anticipating recovery and future employability to avoid causing relapses [12].

In the UK, most employees with ME/CFS fall under the Disability Discrimination Act 1995 [61]. Most other western countries will likely have a similar Act in place. This Act requires that an employer should make ‘reasonable adjustments’ to the workplace and to working practices, so that a disabled or chronically ill employee is not at a disadvantage compared to abled bodied employees and is able to work despite his or her disability. Workplace adjustments that fall under this disability act, could include: changing location of work, working from home, limiting working hours, reducing workload and limiting or reducing physical tasks [60,61,62]. Small modifications to the working environment can make a big difference for ME/CFS patients. Examples of such modifications might be creating a quiet area to rest without being disturbed or the use of an allocated parking space near the entrance of the building [61,62].

People with ME/CFS often feel under pressure to continue working when they first become ill or when their symptoms worsen. Unfortunately, trying to push through this illness is counterproductive, potentially causing longer sickness absences and slower recovery. Returning to work after a period of illness with ME/CFS requires a much more gradual approach than most other phased returns and can require a year or more instead of weeks or months. A return to previous hours within eight weeks, as happens with some other illnesses, is likely to be counter-productive. A slow and gradual return tailored to the individual and his symptoms is more likely to be sustainable without leading to relapses which can cause long term sick leave. It is important that (time to) travel to/from work, is incorporated and taken into account in the work rehabilitation programme [61]. It can be difficult for employees with ME/CFS to maintain a consistent level of working, because of the fluctuating nature of the illness, whereby symptoms can also vary from day-to-day. This can be frustrating and challenging for all parties involved including the employee with ME/CFS. In some cases, flexible working hours might be the solution to that [61,62].

NIVEL, the Netherlands Institute for Health Services Research, published a report (*n* = 412) about ME/CFS [104]. They found that 20.7% were working a mean of 20 h per week, 6.1% were going to school or studying and 71.0% were partially or fully medically retired from work due to ME/CFS. According to research by TNO [2] (*n* = 924), another independent Dutch research institute, 34% were fully and 22% were partially medically retired. The most important problems interfering with work were severe and disabling chronic fatigue, concentration problems/cognitive dysfunction and/or muscle pain (for more information about this TNO report, see earlier). According to the findings by NIVEL, only 22.5% of those who were working, were working more than 24 h per week. The report found that there are a number of important things according to the respondents who were working, which had enabled them to (return to) work. The most important thing for 92% was support in finding the right balance between work and spare time and support and cooperation from the employer to enable patients to continue to work (84%). Other things that respondents found important were the following: supplying information about ME/CFS to colleagues and superiors (62%), changing tasks (61%) and reducing the number of hours they had to work (61%); more rest periods during working times (60%) and the availability of a special rest place at work (45%); working from home (52%), individual support and coaching in general (51%) and by an occupational health physician in particular (44%); adjustments to working conditions (furniture, physical aids) (38%) and a regulation or provision for commuting to work (36%).

### 4.4. Medical Retirement

In cases when incapacity is prolonged, work rehabilitation is impossible or unsuccessful and prognosis appears to be poor, then medical retirement might be the only option. The occupational physician may then be asked to advise on this if the employee is covered by a company pension scheme which makes this provision. Qualifying criteria inevitably vary, although permanent inability to undertake normal duties for reasons of ill health is a common requirement [60,61,62].

## 5. CBT and GET and Work Outcome

A systematic review by Whiting et al. [106] concluded that many studies of behavioural therapies in ME/CFS do not use outcomes that are relevant to patients. Examples of outcomes that would be relevant to them, according to Whiting et al., but also according to a systematic review by Smith et al. [107], would be quality of life, objective outcome measures—like the actometer or the six-minute walk test—and employment and disability status. CBT and GET studies that reported on work outcomes are presented in Table 4. 

Akagi 2001 et al. [108], concluded that CBT was effective and that those who worked had increased from 15 to 27 (*n* = 94). However, 10 of those 27 were actually on sick leave, 5 of those 27 were unemployed and 77% of those working changed occupation due to their illness. Also, the dropout rate was 46% (43/94). Moreover, it was a non-randomised study without a control group and patients were selected if they satisfied the Oxford criteria or criteria for neurasthenia yet all of them were classified as having ME/CFS.

Dyck et al. [115] was a case study (*n* = 2) of a multidisciplinary programme including 30 minutes of fitness twice a week. Such a workload would exclude most patients with ME/CFS. Fulcher and White (*n* = 66) [117] created a study that used the Oxford criteria. 39% were working or studying at least part time at trial entry, compared with 47% after treatment. However, as found by the reanalysis of the Cochrane exercise review for ME/CFS [159], there were a number of issues with this study. Participants in the exercise group had sessions of five to fifteen minutes, increasing to a maximum of thirty minutes, at least five days a week. Such a workload would exclude most patients with ME/CFS. Moreover, the normal fitness scores (VO2max) at baseline in the GET group (31.8) cast further doubts about the diagnosis as this score is well above the score for mildly impaired ME/CFS patients (22.1) according to VanNess et al. [160]. It is also well above the threshold of impairment (25) according to the American Medical Association [161]. Moreover, patients with a common symptom of ME/CFS (sleep disturbances) were excluded yet patients on full dose antidepressants were not. All this together raises serious concerns about whether this was in fact a trial for patients with ME/CFS. Finally, there was an important difference of the fitness of the GET group at baseline compared to the control group (VO2max score 31.8 versus 28.2).

In a non-randomised non-controlled trial by McDermott et al. [128], 9.2% (9/98) returned to work full time and 6.1% (6/98) part-time after a lifestyle management programme. This programme used the principles of CBT and graded activity for ME/CFS within a biopsychosocial framework [113] with pacing as the core strategy [128]. Pacing is an illness management strategy to stay within one’s energy envelope which has been practiced by patients for a long time as a strategy to try and prevent relapses and optimise the things they can do [162].

A non-randomised non-controlled trial of six patients that tested the efficacy of group CBT by Saxty et al. [138], found that the two people who had been working part time had increased their hours and the one patient who was working full time continued to do so. Wittkowski et al. [152] conducted a non-randomised non-controlled trial of group CBT that also only included six patients. Two of them dropped out, one patient returned to full time employment while another worked part time on a phased return. Scheeres et al. [139] was another non-randomised non-controlled study in which “relatively many patients (62%) had a paid job” at baseline according to the authors. The number of contract hours after CBT decreased from 16.2 to 14.9 but the number of hours worked increased from 9.4 to 11.4 per week. 

Marlin et al. (1998) [125] was a non-randomised study in a privately funded clinic of a multidisciplinary intervention consisting of medical treatment if needed, pharmacological treatment of comorbid defective or anxiety disorders and CBT for ME/CFS. 50% of the participants in the treatment group were treated with full-dose antidepressant, which suggests that they were suffering from depression. There was 25.5% (13/51) and 21.6% (11/51) in the treatment group and 0% and 5% in the no treatment control group had resumed work at the end of treatment and follow-up, respectively. Many in the no treatment group had refused to take part in a behavioural intervention programme. Also, 69% (49/71) were lost to follow up.

Friedberg et al. [116] was set up to assess the efficacy of behavioural self-management (CBT delivered by a booklet and audio CDs) in severe ME/CFS. The authors concluded that there was significantly reduced fatigue at three months but not at twelve-month follow-up compared to the no treatment control group (usual care). Also, that it appeared to be less effective in comparison to findings reported for higher functioning groups by other trials. The trial found that behavioural self-management did not lead to objective improvement (actigraphy, step counter and six-minute walk test). At baseline, 15.3% (21/137) worked full time and 21.2% (29/137) part-time or halftime. No employment data is available from follow up. The high rate of participants working at baseline together with the distance walked during the six-minute walk test (336 m), raises serious concerns about whether this was in fact a trial for patients with severe ME/CFS.

Other trials of behavioural interventions that provided employment data at baseline but not at follow-up, were conducted by for example Hlavaty et al., Lopez et al., Schreurs et al., Vos-Vromans et al. and Wearden et al. [118,124,140,142,145].

Trials by Bazelmans et al., Jason et al., O’Dowd et al. and Prins et al. [109,122,130,131], with follow-up ranging from 6 to 14 months, found no statistically significant difference in employment status between the treatment and control group at follow-up. This was also found by Deale et al. [114] at 5-year follow-up.

More patients had resumed work at 4- and 12-months follow-up in the no treatment control group compared to the group that was treated with CBT delivered by GPs in a trial by Huibers et al. [119].

An evaluation of the efficacy of CBT in the Netherlands by Koolhaas et al. [123] found that after CBT, patients worked five hours per week less and the percentage of patients who were able to work, had decreased from 41% to 31%.

In the PACE trial (*n* = 641) by White et al. [148], lost employment remained the same (84%) after CBT and increased from 83% to 86% after GET. The number of participants on income benefits increased from 10% to 13% (CBT) and from 14 to 20% (GET); disability benefits increased from 32% to 38% (CBT) and from 31% to 36% (GET); payments from income protection schemes or private pensions increased from 6% to 12% (CBT) and from 8% to 16% (GET) [150]. Evaluation of the efficacy of CBT and GET in the Belgian CFS knowledge centres (*n* = 655) showed that employment status decreased from 18.3% to 14.9% and sickness allowance status increased from 54% to 57% [141].

Collin and Crawley [111] analysed the efficacy of treatments provided by 11 CFS/ME specialist services in the UK (*n* = 952). These services treated patients with CBT, GET, a combination of both or activity management which was more effective in fatigue reduction at 12 months follow-up than CBT and GET. Also, there was no change in employment situation after treatment in the NHS clinics in 47.2 % cases. 18.0% were able to return to work or increase their hours and 30.0% stopped working or reduced their hours because of ME/CFS. Therefore, the net effect was that 12% stopped working or reduced their hours after NHS treatment.

## 6. Discussion

A large supplier of nationwide occupational health services had questions about medical retirement for ME/CFS, how ME/CFS affects a person’s ability to work and what can be expected in terms of recovery. Yet they were unable to find an article in the medical literature addressing these questions. We were also unable to find such an article. Therefore, we reviewed the literature to see if we could answer these questions.

The name, chronic fatigue syndrome, has had a huge impact on the medical, scientific and patient communities—how it is viewed and how patients are treated by the medical profession [163]. That together with the fact that most ME/CFS patients look well and have no outward signs of illness, combined with the lack of training in medical school and during post graduate education, means that many doctors are not aware of the severity of ME/CFS [164] or that 25% are too ill to leave their homes [53]. Nor are they aware of the fact that the quality of life in ME/CFS is worse than in other severe illnesses like MS, lung cancer, chronic renal failure or stroke [165]. 

Most cases tend to start as an unremarkable viral infection. However, instead of recovering, patients begin to experience profound muscular (and cognitive) fatigue—for example heavy legs—following activities which were previously completed without difficulty. Also typical is an abnormally prolonged delay in the restoration of muscle (and brain) power [166]. Consequently, people with ME/CFS are often unable to engage in economically productive work and typically request sick leave as a solution to their health crisis [167]. Prior to developing ME/CFS, most patients were healthy, sporty and active [4]. There is no diagnostic test, therefore diagnostic criteria are used to diagnose ME/CFS. Over the last 35 years and especially in the last 5 to 10 years, many different biological abnormalities have been found in patients with ME/CFS distinguishing them from healthy controls [22]. Due to these, the Institute of Medicine—now called the American National Academy of Medicine—concluded in 2015 that ME/CFS is a severely debilitating chronic multisystem disease [23]. A great deal more is known today about the underlying biology of ME/CFS but unfortunately, we do not have a diagnostic test yet. However, a recently published small study of 20 cases and 20 controls, reported that a test they had developed involving a nanoelectric chip, which is capable of measuring minuscule energy changes in cells in the blood to gauge their health when exposed to stress. In this case salt, was able to distinguish between cases and controls with 100% certainty [168]. A much larger study is now needed to confirm the accuracy of this test. Not only to distinguish cases from healthy controls but also to distinguish them from other fatiguing illnesses. 

Growing awareness of the underlying biological underpinnings has created increased international awareness and interest in the illness. This will accelerate research and the finding of a diagnostic test and effective pharmacological treatment [22]. However, we will have to continue to rely on diagnostic criteria to diagnose the illness as has been the case so far until such a test becomes available.

Just like with most other illnesses, illness severity can vary between patients. Mildly affected patients have a substantial activity reduction according to the Fukuda criteria [18], and at least a 50% activity reduction in comparison to before they fell ill according to the 1988 CDC criteria [105] and the 2011 International Consensus Criteria [20]. Unfortunately, postexertional malaise (PEM), the main characteristic of the disease, is not a requirement for diagnosis according to the Oxford criteria [13] which are primarily used in the UK. Moreover, it is only an optional requirement according to the Fukuda criteria [18], the most commonly used criteria to diagnose ME/CFS. PEM is compulsory for diagnosis according to newer diagnostic criteria—the Canadian Consensus Criteria and its revised version, the International Consensus Criteria [12,20] as can be seen in Table 1. The consequence of using criteria that do not require the main characteristic of the disease to be present is that in a substantial number of cases, as discussed earlier, patients do not suffer from ME/CFS but they have a self-limiting illness [15,16] or a disease which in many cases would be treatable if patients had gotten the right diagnosis. The combination of a lack of a diagnostic test, using different diagnostic criteria and the lack of adequate training about this illness in medical school has led to 2 problems. First of all, up to 50% of patients diagnosed with ME/CFS have an alternative explanation for their symptoms [25,26]. Many of the alternative diagnoses are currently treatable which would mean that many patients could go back to work if they would get the right diagnosis and treatment. The diagnosis of ME/CFS should be reconsidered if none of the following key features are present:: post-exertional fatigue or malaise, cognitive difficulties, sleep disturbance (unrefreshing sleep or reversal of sleep pattern) and chronic pain [8]. The diagnosis should also be reconsidered if patients deteriorate or get new symptoms. Secondly, according to estimates, around 90% of patients affected by ME/CFS are not diagnosed with the disease. Improving diagnosis and optimizing management can have significant economic and public health consequences [9]. In particular because shorter illness duration is a significant predictor of sustained remission, and thus early detection of ME/CFS is of utmost importance [34].

A meta-analysis by Franklin et al. [169] of 32 studies found that ME/CFS patients have a substantially reduced VO2peak compared to healthy sedentary controls. If occupational health physicians have doubts about the legitimacy or the severity of the disease in a specific case then this physiological abnormality, together with postexertional malaise, can be detected by two-day cardiopulmonary exercise testing (CPET) using the protocol of the Workwell Foundation [170]. The downside of CPET in ME/CFS however is that it is expensive, it can lead to relapses and severely affected patients are too ill and disabled to do it. Alternatively, in such a case, it could be worthwhile considering a second opinion by a ME/CFS literate medical doctor.

### 6.1. What Can Be Expected If a Patient Is Diagnosed with ME/CFS?

A comprehensive review of the literature on the natural course of ME/CFS in adults showed that the illness runs a chronic course. A progression or worsening of symptoms is seen in 10 to 20% of cases and overall the prognosis in terms of return to work is poor. Also, only 5% will recover [10]. In clinical trials of ME/CFS, the term recovery often reflects less than full restoration of health. A more appropriate and accurate label for this would be clinically significant improvement [164]. However, it’s not only clinical trials that suffer from this problem. Brown et al. [65] found that adults who labelled themselves as recovered from ME/CFS, showed significant impairments on 21 out of 23 outcomes compared to healthy controls. Moreover, on 17 of those 23 outcomes, the impairments were the same as for those who were still ill with ME/CFS, which suggests that patients adapt to their impairments instead of recover from them. A working group, reporting to the Chief Medical Officer (CMO) for England [55], concluded that most of those who feel recovered stabilise at a lower level of functioning than that before their illness. Consequently, even a recovery percentage as low as 5, might well be too optimistic. 

Contrary to typical patterns of chronic disease, where the most severe cases present to medical professionals, severe cases of ME/CFS are less likely to do that [28] due to being bedridden and too ill to attend. Several studies have shown that the prognosis for patients with severe ME/CFS, including young patients with severe ME/CFS, is worse than for ME/CFS in general. Early management of the illness appeared the most important determinant of severity [54]. Dr Melvin Ramsay [36], the infectious disease specialist who witnessed and documented the outbreak of ME in the Royal Free Hospital in London in 1955, wrote the following about that: “The degree of physical incapacity varies greatly, but the dominant clinical feature of profound fatigue is directly related to the length of time the patient persists in physical effort after its onset; put in another way, those patients who are given a period of enforced rest from the onset have the best prognosis.” In the absence of effective treatment, this is the only thing that occupational health physicians and other doctors can do in the beginning of the illness, to try and prevent chronic and severe physical incapacity, long-term sick leave and medical retirement later on in the course of the disease.

### 6.2. Factors Predictive of a Worse Outcome

Illness severity is strongly implicated in a poor prognosis for ME/CFS [54]. Those who are more fatigued experience a greater number of somatic symptoms and an increase in functional limitations. These factors make it more difficult to recover from ME/CFS [41]. A systematic review by Cairns and Hotopf [10] found that having a comorbid psychiatric disorder at baseline was associated with poorer outcome and Vercoulen et al. [49] found the same for cognitive factors. Psychosocial factors show little relationship to recovery [171]. Smoking and personality are not risk factors [172]. Neurotic traits are more frequent among the less severely ill. Conscientiousness is not related to severity [54]. Patients with ME/CFS and Fibromyalgia (FM)—FM is a comorbidity in 50% to 60% of cases—are three times more likely to become non-improvers than those without FM [44]. Patients who are more ill and have comorbidities are less likely to be able to work than those with milder ME/CFS without comorbidities [3]. While there is good reason to suggest that a positive attitude will help in the prognosis of any disease, including ME/CFS, there is little empirical evidence to support the assertion that attitudes, behaviour or underlying personality have a major role in determining outcomes [54]. Men, people in older age groups, and people who have been ill for longer are more likely to have ceased employment due to their illness. These factors are predictive of a worse outcome [45,46]. Poorer outcome is also predicted by increased pain and worse physical functioning at onset [45]. Disability was the main independent predictor of discontinuation of employment in a study of employees on long-term sick leave due to fatigue [56]. The NHS database findings suggest that people with ME/CFS continue in employment despite the primary (fatigue and pain) and secondary effects (depression and anxiety) of ME/CFS. Instead, loss of physical capacity is the ultimate arbiter of inability to continue working [46]. The prognosis is better if patients developed ME/CFS during an outbreak [173], after glandular fever/mononucleosis [52] or after Giardia enteritis [35,79].

### 6.3. Employment Status 

The inability to work due to ME/CFS is high [59]. Between 27% and 65% of CFS patients are reported not to be working, and less than a third of patients are estimated to resume employment within three years after diagnosis, as found by a systematic review [10]. Many who improve, experience the majority of their improvement relatively quickly [55]. Van der Werf et al. [94] found that after a follow-up period of 12-months, spontaneous recovery was rare and only occurred in patients with an illness duration of less than 1.5 years. A prospective study by Vercoulen et al. [49] followed up 298 patients with a relatively long duration of complaints—median 4.5 years—for 18 months. They found no improvement in employment and benefit status. This despite the fact that the study included 51 patients with an illness duration of less than two years, who according to the same study are more likely to improve. Nine-year follow-up of a Danish group of CFS patients, showed that recovery and substantial improvement are uncommon and that patients as a group neither improved nor deteriorated since diagnosis [33].

Studies that reported on employment status found large differences between ME/CFS patients and healthy controls (see Table 2) but also between ME/CFS, MS, depression and healthy sedentary controls [80]. These data are often from small(er) studies, larger studies show the following. Castro-Marrero et al. [3] (*n* = 1757) found that 62.8% were unemployed due to ME/CFS and 25.6% were employed. The rest had never worked. Collin et al. [46] who analysed the NHS database (*n* = 2170) found that 41% were employed and 50% had discontinued work due to ME/CFS which is similar to the 54% found by a systematic review by Ross et al. [59]. TNO, an independent Dutch research institute [2] (*n* = 924) found that 30% were working; 7% had never been on long-term sick leave and 23% had been able to go back to work after long-term sick leave but they were working less hours. They were also less often involved in management and more often did sedentary work behind a computer. Nivel, another independent Dutch research institute [104] (*n* = 412), found similarly high rates of patients unable to work due to their ME/CFS. It also found that only 1/4 of patients who worked, were able to work more than 24 h a week. Reports by ME Associations from around the world [98,99,100,101,102] also highlight the large number of patients who are unable to work due to ME/CFS (see Table 3).

### 6.4. Medical Retirement 

In cases where incapacity is prolonged, work rehabilitation is impossible or unsuccessful and prognosis appears to be poor, then the occupational health physician may be asked to advice on the possibility of retirement on the grounds of ill health if an employee with ME/CFS is covered by a company pension scheme which makes this provision. Qualifying criteria inevitably vary, although permanent inability to undertake normal duties for reasons of ill health is a common requirement [60]. Important aspects of a work capability and functional capacity assessment are the influence of symptoms such as pain, fatigue and cognitive problems on the ability to work, bearing in mind that cognitive problems, together with physical problems are often the reason why patients stopped working [46]. It is estimated that between 74% and 95% of ME/CFS patients have some type of cognitive deficit [174,175] which are cited as some of the most disruptive and functionally disabling symptoms of ME/CFS [114]. Studies of objective neuropsychological functioning in ME/CFS consistently document impairment in information-processing speed, auditory attention and memory [176]. In addition, Chu et al. [68] found that cognitive symptoms present at the beginning of the illness tend to persist, declining by only 4–10%.

Although many patients are eventually retired, such action should be a last resort. On the other hand, the prognosis for recovery and substantial improvement that enables a return to work is poor if patients have been off work for 2 to 3 years. This was confirmed by the Inspectorate Work and Pay of the Dutch Ministry of Work and Social Affairs [177]. This Inspectorate concluded that if patients have been on long-term sick leave for two years or more and treatment with CBT did not make a difference, then the prognosis for a return to work is poor.

If employees are mildly affected yet unable to work and the occupational health physician is reluctant to award medical retirement than it seems reasonable to award retirement benefits for a limited time followed by a case review in six months to a year. Long-term compensation to secure the socioeconomic position does not inhibit return to work, but may be an essential contributor to becoming employed later on [52]. The reason for this might be that it prevents patients from going over their limits if they would be forced to do work which they are unable to do which might seriously disrupt the naturally occurring recovery or improvement process in persons with ME/CFS.

### 6.5. Strengths and Limitations of This Review 

Limitations of this review are caused by the use of different selection criteria by the studies in the review, so that patients might have been included who do not have ME/CFS. Also, by the variability in follow-up periods. Furthermore, some studies are prospective whilst others are retrospective. In some studies patients were seen by physicians to check the diagnosis, other studies relied on questionnaires only. Studies did not consistently report about work status at both baseline and follow-up. Nor did they consistently describe employment status as full-time or part-time, previous or new work, or duration before returning to work. Studies also only measured outcomes at baseline and one follow-up moment but not more frequently. Therefore, in those studies it was impossible to see whether self-rated improvement or recovery and return to work was maintained or temporary.

Despite these limitations, one conclusion supported by all studies is that ME/CFS patients who fulfil strict diagnostic criteria, have worse prognosis compared to patients fulfilling lenient criteria and that the prognosis in general is poor. Reports by two independent Dutch research institutes, the large Spanish study by Castro-Marrero et al., the evaluation reports of the Belgian CFS clinics, the British NHS CFS clinics and the NHS database [2,3,45,46,104,111,141] provide detailed analyses about employment status in CFS adding to the strength of the evidence gathered by this review. Another strength of this review is that 38 CBT and/or GET studies that reported on work status were reviewed. Previously, a systematic review by Ross et al. (2004) [59] reviewed 4 CBT and/or GET studies that did so. Systematic reviews by Cairns and Hotopf (2005), Castro-Marrero et al. (2017), Chambers et al. (2006), Malouff et al. (2007), Smith et al. (2015) and Whiting et al. (2001) [10,106,107,178,179,180], a systematic review and meta-analysis by Marques et al. (2015) [181] and a meta-analysis by Castell et al. (2011) [182] did not review this. 

### 6.6. Do CBT and GET Restore the Ability to Work in ME/CFS? 

An influential systematic review by Cairns and Hotopf [10] concluded in 2005 that because there is increasing evidence for the effectiveness of CBT and GET, that “Medical retirement should be postponed until a trial of such treatment has been given.” Consequently, many patients in The Netherlands have been forced to undergo these treatments and illness benefits and medical retirement were often not awarded if patients refused to do so. The Dutch Health Council concluded in March 2018 that ME/CFS is a serious and chronic multisystem disease and that CBT and GET are not adequate medical treatments for ME/CFS [24]. It also concluded that patients should not be forced to undergo these treatments. However, the chairman of the Dutch Association of Insurance Physicians said in a recent interview in a Dutch medical journal [183] that he did not agree with this. He was also urging insurance physicians to question patients’ recovery behaviour if they refused to be treated with CBT and GET and to force patients to undergo these treatments. In the Netherlands the more than 700 insurance physicians of the UWV (Uitvoeringsinstituut Werknemersverzekeringen or Employee Insurance Agency) [177] decide if employees will be granted (temporary) medical retirement.

This raises the question if these treatments restore the ability to work. To answer this question, we analysed studies that tested the efficacy of CBT and/or GET and reported on employment outcomes (see Table 4). One of these studies was a study by Prins et al. (2001) [131], the largest CBT trial from the Netherlands (*n*= 278) which found that CBT does not improve employment status compared to doing nothing. Another important study was the PACE trial (*n* = 641) [148], the largest CBT and GET trial ever conducted. The efficacy of these treatment has also been assessed in real life outside of clinical trials, in the Belgium CFS knowledge centres (*n* = 655) and the NHS CFS clinics (*n* = 952) [111,141]. These evaluations, just like the PACE trial itself, showed that CBT and GET do not improve employment and illness benefit status. As a matter of fact, both deteriorated. After treatment, more patients were unable to work and more were receiving illness benefits. Also, a systematic review by Ross et al. [59] concluded in 2004 that CBT and GET did not prove effective in restoring the ability to work. Chambers et al. and Castro-Marrero et al. [178,179] documented this conclusion by Ross et al. in their systematic reviews in 2006 and 2017, respectively. Consequently, being treated with CBT and GET should not be a requirement to be eligible for medical retirement. Moreover, there is also no point in questioning patients’ recovery behaviour or forcing them to undergo these treatments.

## 7. Conclusions

Myalgic Encephalomyelitis/Chronic Fatigue Syndrome leads to severe functional impairment and work disability in a considerable number of patients. The majority of patients who manage to continue or return to work, work part-time instead of full time in a physically less demanding job. The prognosis in terms of returning to work is poor if patients have been on long-term sick leave for more than two to three years. Being older and more ill when falling ill are associated with a worse employment outcome. Cognitive behavioural therapy and graded exercise therapy do not restore the ability to work. Consequently, many patients will eventually be medically retired depending on the requirements of the retirement policy, the progress that has been made since they have fallen ill in combination with the severity of their impairments compared to the sort of work they do or are offered to do. However, there is one thing that occupational health physicians and other doctors can do to try and prevent chronic and severe incapacity in the absence of effective treatments. Patients who are given a period of enforced rest from the onset, have the best prognosis. Moreover, those who work or go back to work should not be forced to do more than they can to try and prevent relapses, long-term sick leave and medical retirement.

## Figures and Tables

**Table 1:** Summary of case definition criteria.

Oxford Criteria (1991) [13]	Fukuda Criteria (1994) [18]	Canadian Consensus Criteria (2003) [12]	International Consensus Criteria (2011) [20]
Chronic disabling fatigue for ≥ 6 months during which it was present for > 50% of the time. No other symptoms required	Chronic fatigue of ≥ 6 months At least 4 of the following symptoms: Impaired memory/concentration Sore throat Tender cervical or axillary lymph nodes Muscle pain Multi joint pain New headaches Unrefreshing sleep Post-Exertional malaise	A minimum of 6 months of: fatigue post-exertional malaise and/or fatigue sleep dysfunction pain Also have two or more neurological/cognitive manifestations and one or more symptoms from two of the categories of autonomic, neuroendocrine, and immune manifestations	A patient will meet the criteria for postexertional neuroimmune exhaustion (A), at least one symptom from three neurological impairment categories (B), at least one symptom from three immune/gastro-intestinal/genitourinary impairment categories (C), and at least one symptom from energy metabolism/transport impairments (D). A. Post exertional neuroimmune exhaustion (PENE): compulsory. Characteristics: Marked, rapid physical and/or cognitive fatigability in response to exertion, which may be minimal such as activities of daily living or simplemental tasks, can be debilitating and cause a relapse Postexertional symptom exacerbation Postexertional exhaustion Recovery period is prolonged Low threshold of physical andmental fatigability (lack of stamina) results in a substantial reduction in pre-illness activity level. B. Neurological impairments At least one symptom from three of the following four symptom categories: Neurocognitive impairments (Difficulty processing information, Short-termmemory loss) Pain (Headaches, significant pain). Sleep disturbance Neurosensory, perceptual and motor disturbances C. Immune, gastro-intestinal and genitourinary impairments (symptoms from at least 3 of the following categories): Flu-like symptoms Susceptibility to viral infections with prolonged recovery periods Gastro-intestinal tract symptoms Genitourinary symptoms Sensitivities to foods, medications, odors, or chemicals D. Energy production/ion transportation impairments (symptoms from at least 1 of the following categories): Cardiovascular symptoms Respiratory symptoms Loss of thermostatic ability Intolerance of extremes of temperature Severity: Mild (an approximate 50% reduction in pre-illness activity level) Moderate (mostly housebound) Severe (mostly bedridden) Very severe (totally bedridden and need help with basic functions).

**Table 2:** Work-related outcomes and naturally occurring improvement rate.

Study	Criteria	*n*	Mean Age in Years	Illness Duration at Baseline	Length of follow-up (FU)	Works Status	Rate of Improvement
Andersen et al. (2007) [33]	Meeting both CDC 1988 and Fukuda	34	46.4 at diagnosis	4 yrs	9 yr	76.5% (26/34) medically retired; 1 worked full time in physically less demanding job, 2 worked part-time, 3 were freelance + on disability payments	As a group patients had not improved; 6% recovered & 10% had received other diagnosis
Assefi et al. (2011) [63]	Fukuda	555 (fatigue study, 207 CFS patients)	38.2	4.4 yr	No FU	Of the CFS patients, 61% worked, 44% worked less hours, 29% lost jobs due to illness, 30% received illness benefits; 23% changed jobs due to illness, 30% took significant pay cut	No FU
Behan et al. (1985) [64]	Unclear	50	37	5 yrs	No FU	4 of the 5 doctors and all 8 nurses were unable to continue work; the medical student withdrew from his course for a yr. No employment data provided for the other 37 patients	The illness was chronic in 37 patients but had a relapsing and remitting course in 13.
Bombardier and Buchwald (1995) [38]	CDC 1988	498 (fatigue study, 226 CFS patients)	38.1	5.2 yrs	1.5 yrs	CFS patients at FU: 40% unable to work at all, 20% unable to work full-time, 22% decreased work performance, 16% increased work performance, 11% resuming full time and 13% part-time work.	2% recovered, 24% had worsened, 12% were unchanged, rest improved slightly to significantly.
Brown et al. (2012) [65]	Bell and Bell 1988	35 (25 CFS + 10 healthy controls HC)	37	25 yrs	25 yr follow-up of patients who fell ill as adolescents; average age at illness onset 12.1 yrs [66]	Full-time employment: 90% HC, 71.4% CFS. CFS: working part time 11.4% and 16.4% on disability	80% remitted yet still showed more impairment on 21 of 23 outcomes compared to healthy controls and on 17 of 23 outcomes there was no difference with those who maintained a CFS diagnosis
Buchwald et al. (1996) [67]	1998 CDC	431 (fatigue clinic patients including 185 CFS) + 99 HC	39	4.7 yrs	No FU	Employed: CFS 46% (part-time and full time) vs. 91% HC	No FU
Castro-Marrero et al. (2017) [3]	Meeting both Fukuda and Canadian criteria	1757	47.7	10 yrs	No FU	62.8% unemployed, 25.6% employed, 11.6% never worked	No FU
Chu et al. (2019) [68]	Fukuda	200, no controls	53.7	Unclear	2 yrs	At baseline, 47% permanently disabled; 15% worked >30 h/week	Response rate: 75% (150/200); 4% improved, 96% no improvement
Ciccone et al. (2010) [44]	Fukuda	94 (women only, no controls)	41.6	5.9 yrs	Biannual telephone surveys over a period of 2.5 yrs	Employed: 50.8% improvers, 29.0% nonimprovers. Disabled: 41.3% improvers, 71.0% nonimprovers	Response rate: 63.5% (94/148); 67% improved but were still far short of recovery
Clark et al. (1995) [37]	1988 CDC	98, no controls; chronic fatigue study, 19 CFS patients	39.9	5.5 yrs	2.5 yrs	Employment status not mentioned	Response rate: 79.6% (78/98); of the CFS patients 7 (37%) recovered and 12 (63%) did not recover
Claypoole et al. (2001) [69]	Fukuda	29 twin pairs (monozygotic twins and their healthy siblings)	41.2	7.2 yrs	No FU	Employed: 43% CFS, 90% HC	24% dropped out.
Collin et al. (2011) [46]	Fukuda	2170	38.6 women, 41.4 men	35 mo currently employed, 48 mo employment discontinued	Single measurement, no FU	40.7% were employed, 50.1% had discontinued work due to CFS	No FU. Employment status recorded for 1991 patients (91.8%).
Garcıa-Borreguero et al. (1998) [70]	Fukuda	82 (41 CFS and 41 healthy unrelated neighbours)	37.6 CFS, 38.4 healthy neighbours	5.5 yrs	No FU	Vocational disability: 17.1% partial, 56.1% full CFS, not applicable in healthy neighbours	No FU
Hill et al. (1999) [47]	1988 CDC	23 ("severe" subset of CFS patients)	35	2.4 yrs	3.4 yrs (FU at 1.6 yrs and also at 3.4 yrs)	Employed at baseline: 5 full-time and 1 part time; 2 returned to part-time work at 1st follow-up and 1 of them became disabled again	4% recovered; majority showed no improvement
Huibers et al. (2006) [71] and Leone et al. (2006) [56]	Fukuda	151 fatigued employees (52 with CFS like cases at baseline)	43.9	35.0 mo CFS like cases (at baseline)	4 yrs (FU at 1 yr and 4 yrs)	Work disability CFS like cases: 41% baseline, 20% at 12 month FU, 27% at 4 yr FU. At final follow-up, 59.6% were on sick leave, full or partial work incapacity, unemployed or retired	Response rate: 84% (127/151). 40% went on to meet CFS criteria at follow-up; 16.9% developed a CFS like status during the 4 yrs and 57% still met criteria for severe fatigue.
Jason et al. (2008) [72] study 1	Fukuda	79 (32 CFS vs. 47 HC)	37	Unclear	No FU	Working full time: 33.3% (CFS) vs. 86.7% (HC); Part-time: 19% vs. 6.7% Unable to work due to illness 42.8% vs. 0%	No FU
Jason et al. (2008) [72] study 2	Fukuda	114 (no control group)	42	Unclear	No FU	Working: 26.4% part-time and 25.3% full-time. 76% had to cut down on their work, 49.4 % were receiving disability or were unemployed due to CFS	No FU
Jason et al. (2011) [41]	Fukuda	213 (study included 32 with CFS and 47 HC)	36.8 CFS 41.4 HC	Unclear	10 yrs	At baseline: on disability 20.8% CFS and 9.1% HC Working part time: 8.3% CFS and 13.6% HC; full-time: 37.5% CFS and 68.2% HC. No employment data provided for follow-up.	86% of CFS patients followed up. Over time the CFS group remained rather ill
Johnston et al. (2016) [28]	CFS diagnosis by their primary physician	535 (30.3% Fukuda cases; a further 32.0% met both Fukuda and ICC; 23.2% CF; 14.6% received other diagnosis)	46.4	14.5 yrs	No FU	Fukuda: 12.4% working full-time, 27.8% part-time; receiving disability 30.3%, unemployed 27.8%; ICC: 9.8% working full-time, 28.0% part-time, 34.7% receiving disability, 25.4% unemployed	No FU
Levine et al. (1992) [73]	Postviral fatigue syndrome defined on the basis of severe persistent fatigue	31 patients following one of four outbreaks in USA	Incline Village + Truckee 40.7; Yerington 31.1; Placerville 41.1	Unclear	3 yrs	No employment data	Response rate: 90.3% (28/31). At 2 years 46.2% (12/26) functioning without limitation, after 2 years almost all study objects were back to pre-illness activity
Lin et al. (2011) [74]	Fukuda	500 (264 chronic fatigue, 112 CFS, 124 HC)	35.8	CFS patients: 53% onset age 25 or later, 15% age 24 or earlier, 32% age unknown	No FU	Working during the last 4 weeks: 71% CFS vs. 95% healthy controls	No FU
Lloyd et al. (1994) [75]	Lloyd 1988	25 (12 male CFS patients, 13 male HC)	33.5	60 mo	No FU	41.7% (5/12) were working on a limited part time basis (CFS) vs. 100% HC (full-time); 58.3% (7/12) had stopped working due to CFS	No FU
Lowry and Pakenham (2008) [76]	Fukuda	139	48.3 yrs	11.2 yrs	No FU	24% in some form of employment, 40% on sick leave or disability benefits, 19% retired, 17% divided equally between the categories of student, unemployed (but able to work), or performing home duties	No FU
Matsuda et al. (2009) [77]	Japanese CFS criteria	155	32.7 yrs	54 mo	22.5 mo	At baseline: 47% were working; 42% unemployed and 11% student. No employment data for follow-up	Response rate: 45% (70/155); 12% recovered, 85% had a poor outcome.
McCrone et al. (2003) [78]	Fukuda	141 (fatigue study, 44 CFS)	40 yrs	Unclear	No FU. Service use assessment.	30% lost employment due to illness	No FU
Naess et al. (2012) [79]	Fukuda	58 (CFS after Giardia enteritis; 38 employees, 20 students)	38.0 females and 31.7 males	2.7 yrs	No FU. Assessment 2.7 yrs after falling ill	34.2% (13/38) Of the employees were working part time, 57.9% (22/38) sick leave, 13.2% (5/38) disability pension. 30% (6/20) of the students studied half time and 70% (14/20) too ill to study.	At the time of assessment 16% (9/58) reported improvement, 28% (16/58) no change, and 57% (33/58) slight or significant worsening.
Natelson et al. (1995) [80]	1988 CDC	113 (41 CFS, 19 MS, 17 major depression, 36 HC)	34.4 CFS, 38.3 MS, 41.9 depression, 34.6 HC	Unclear	No FU	Disabled: 56% CFS, 5% MS, 18% depression, 0% HC. CFS patients who could work were unable to do so without limitations	No FU
Nijs et al. (2005) [81]	Fukuda	54	39	68 mo	No FU	Employment rate 95.0% before CFS; currently 29.4% due to CFS; 50% on disability	No FU
Nisenbaum et al. (2003) [34]	Fukuda	65	46	13.0 yrs	91%, 60% and 37% were followed up for 1, 2 and 3 yrs	Employed: 63.1% at baseline, 61.2% at 1 yr, 55.2% at 2 yr and 55.6% at 3 yr FU. Unemployed due to CFS: 16.9% at baseline, 18.4% at 1 yr, 13.8% at 2 yr and 16.7% at 3 yr FU.	57% had a relapsing remitting course; 23.1% received alternative diagnosis, 10% sustained total remission
Nyland et al. (2014) [52]	Fukuda	111 (CFS after mononucleosis)	Mean age at onset 23.7	4.7 yrs at baseline and 11.4 yrs at FU	6.5 yrs	At the time of falling ill 47% were employed and 52% were students. At baseline 8% worked full time, 1% part-time, 13.5% were students, 75% received full sickness benefits. At follow-up 27% worked full time, 28% part-time and 68.5% (63/92) received full or partial disability benefits.	Response rate: 83% (92/111). About half of younger patients experienced marked improvement.
Pendergrast et al. (2016) [53]	Unclear	557 (4 groups of CFS patients: from US 216, UK 103 and two from Norway (N1, 175 + N2, 63)); nearly 25% too ill to leave their homes	US 52.0 UK 45.6 N1 43.4 N2 34.9	Unclear	No FU	On disability: 56.7% US, 30.2% UK, 84.0% N1, 76.2% N2. Working full or part-time: 13.5% US, 37.5% UK, 9.7% N1, 19% N2	No FU
Ray et al. (1993) [82]	Oxford	48 (24 CFS, 24 HC)	38.3 CFS, 40 HC	46.6 mo	No FU	Working full-time: 13% (3/24) CFS, 71% (17/24) HC	No FU
Roche et al. (2005) [83]	Fukuda	47	46.9	10.7 yrs	No FU	Working full-time 14.9%, part-time 14.9%, unemployed 70.2%	No FU
Rowe et al. (2019) [84]	Fukuda (PEM, unrefreshing sleep and cognitive symptoms were also required)	784 (40% started after EBV)	22.5 yrs (mean age 14.8 at diagnosis)	Illness duration prior to diagnosis: 13.6 mo	8 yrs (FU on up to 6 occasions, 2 to 16 yrs after diagnosis)	At baseline, 5% not working or studying; 8% less then part time; 24% more than part-time; 63% full-time. In comparison: similarly aged healthy people: 85% worked/studied full-time, 6% able to work but unemployed	Response rate 81.8% (641/784). Reporting recovery: 38% at 5 yrs and 68% at 10 yrs; 58% reported continuous pattern of illness with fluctuating severity; 5% remained very unwell and 20% significantly unwell.
Russo et al. (1998) [85]	1988 CDC	98 (fatigue study, 27% CFS, increased to 42% at follow-up)	39.9	5.5 yrs	2.5 yrs	Number of subjects not working at enrolment not given; 29.5% returned to work. Unclear how many of those had CFS	Response rate 80% (78/98); unclear how many had CFS; 3% (2/78) fully recovered and 26% of the sample worse
Saltzstein et al. (1998) [86]	Fukuda	15 female patients	41.2	Unclear; 46.7% (7/15) were ill for less than 2 yrs	2 yrs	All were in full-time employment before CFS, at assessment 40% (6/15) worked full-time, 33% (5/15) part-time and 26.7% (4/15) were unemployed	20% were worse or the same; 80% were improved of which 20% (3/15) reported recovery
Schmaling et al, (1998) [87]	1988 CDC	37 (15 CFS, 11 depression, 11 HC); all participants were female	39.4 CFS, 43.1 depression, 45.6 HC	Unclear	No FU	Working: 13% CFS, 64% of depression, 91% HC	No FU
Schweitzer et al. (1995) [88]	Lloyd 1988	77 (47 CFS, 30 HC)	38 CFS, 29 HC	5.0 yrs	No FU	CFS unemployed: currently 49%, before CFS 13%; 47% (22/47) retired from employment as a result of CFS. No employment figures for HC	No FU
Sharpe et al. (1992) [43]	Minimum of six weeks of fatigue	177 (fatigue study, 66% had Oxford defined CFS)	34 yrs	25 mo	1 yr	38% had left or changed their job because of their illness. 73% had days during the past month when they had been entirely unable to work. No baseline data available for comparison	Response rate: 81% (144/177). 13% recovered, 65% were functionally impaired at follow-up and could not walk 100 yards (90 m).
Stoothoff et al. (2017) [89]	Unclear	541	46.3	Unclear	No FU	62.5% on disability, 17.3% worked full or part time. 14% of those constantly getting worse were still working	59.7% described their illness as fluctuating, 15.9% as constantly getting worse, 14.1% persisting, 8.5% relapsing and remitting; and 1.9% as constantly getting better.
Strickland et al. (2001) [90]	1988 CDC	259 (fatigue study after outbreak, 41% had CFS)	47 CFS	10 yrs	10 years after outbreak	No employment data provided	Response rate 47.5% (123/259), 15% of responding CFS patients had recovered
Thomas and Smith (2019) [91]	Fukuda	226	41.7	62.1 mo	3 yrs	At baseline 34% in employment, 49% unemployed, 16% on sick leave, 24% retired or home-makers.	Response rate: 57.5% (130/226); 29% reported some improvement at 18 mo and 3 yrs FU. Recovery: 2% at 6 mo, 6% at 18 mo and at 3 yrs.
Tiersky et al. (2001) [40]	Fulfilling both the 1988 CDC + Fukuda criteria	47	35.5	25.9 mo	41.9 mo	Employment status did not change; 68% were unable to work due to CFS at baseline and FU; those who worked were only able to perform light duty desk work for 3 to 4 h a day but even this amount of work required rest periods	Response rate: 74.5% (35/47). 57% improved, 43% did not. The majority remained functionally impaired overtime. Overall the prognosis appears to be poor.
Tirelli et al. (1994) [92]	1988 CDC	265	35	3 yrs	24 mo	38.5% (102/265) stopped working activities for a period ranging from 3 months to 2 years No other employment data provided.	Response rate: 100%; 3% recovered, 8% substantial decrease in symptoms, in 89% symptoms persisted
Tritt et al. (2004) [93]	Fukuda	429	41.7	Unclear	No FU	37.1% had taken sick leave for more than four weeks in the last 12 months and 56.6% less than 4 weeks; 18.9% (81/429) were on long-term sick leave	No FU
Van der Werf et al. (2002) [94]	Fukuda	79	34.8	1.4 yrs (minimum illness duration 6 mo, maximum 24 mo)	1 yr	75% were in paid employment before illness onset vs. 29% at baseline. No employment data available from follow-up	Response rate: 98.7% (78/79). At FU: 8% no complaints, 38% less complaints, 37% similar, 17% had deteriorated. Spontaneous recovery was rare and only occurred in patients with an illness duration < 1.5 years
Vercoulen et al. (1996) [49]	Oxford	298 CFS patients (comparison data from 53 HC)	39	8.4 yrs (51 patients with illness duration of ≤2 yrs)	18 mo	Employment status at baseline (BL) and at FU: 12% were unemployed; 28% (BL)and 29% (FU) worked; 43% (BL) and 42% (FU) were on sick leave/medically retired and 17% were housewife, retired or at school.	Response rate: 83% (246/298); 3% recovered; 17% improved, 60% remained unchanged and 20% had become worse
Vercoulen et al. (1997) [95]	Oxford	51 CFS, 50 MS and 53 HC	36.3 CFS	5 yrs CFS	No FU	Working: 27% CFS, 28% MS and 47% HC. Invalidity benefits: 43% CFS, 32% MS and 2% HC. Total hours working: 10.4 CFS, 13.3 MS, 35.7 HC	No FU
Vincent et al. (2012) [96]	Fukuda	151 (76 CFS, 75 IF)	38.2 CFS (at fatigue onset)	3.9 yrs CFS	No FU	CFS affected daily activities and work in 95% of cases	No FU
Wilson et al. (1994) [32]	Lloyd 1988	139	42.2	9.2 yrs	3.2 yrs	30% (31/103) patients unable to perform any work at FU and 25% (26/103) were receiving disability benefits because of CFS. No baseline data available for comparison.	Response rate: 74% (103/139); 37% did not improve, 20% could not perform any significant physical activity and 40% no social activity. Only 5.8% (6/103) had completely recovered
Zdunek et al. (2015) [97]	Fukuda	2 groups of CFS patients: USA 162, UK 83	USA 52.0 UK 45.9	Unclear	No FU	Working full or part-time: 11.2% USA, 35.3% UK. On disability: 55.3% US, 35.4% UK.	UK more gradual onset, USA more sudden onset

BL: baseline; CF: chronic fatigue; EBV: Epstein-Barr virus; FU: follow-up; HC: healthy controls; ICF: idiopathic chronic fatigue; IF: idiopathic fatigue; mo: months; MS: multiple sclerosis; yr: year; yrs: years.

**Table 3:** Work status according to patient charities and independent research Institutes.

Study	*n*	Works Status
25% ME Group (2004) [103]	437 severely affected patients	In receipt of state illness benefits 98% and disability living allowance 86%
Bringsli (2014) [98]	1096	50% received temporary disability benefits, 25% were medically retired 5% worked full time, 10% part-time
Chu (2013) (FDA Survey) [99]	623	Disabled and unemployed due to CFS 53.4% and 21.9%; working part-time 7.0% and full-time 5.7%
De Kimpe (2016) [100]	629	71.38% worked > 8 h a week before falling ill with CFS. Due to CFS only 45.79% are able to work. Also, those who are able to work: > 40 h decreased from 14.8% to 0.8%; 32 to 40 h decreased from 29.7% to 1.6%; 24 to 32 h decreased from 13.67% to 2.34%; 0 to 8 h increased from 1.43% to 27.98%.
Emerge Australia (2018) [101]	610	74% had to stop working due to CFS, this usually occurred around one yr after the onset of symptoms.
ME Association (UK) (2015) [102]	1428	Net increase in disability benefits of 10% after CBT, 13% GET and 1% after pacing
Nivel (2008) [104]	412	71.0% are (partially) medically retired due to CFS. 20.7% worked, mean 20 h/week; 15% worked > 32 h/week.
TNO (2005) [2]	924	30% were working; 7% had never been on long-term sick leave and 23% had been able to go back to work after long-term sick leave but they were working less hours. They were also less often involved in management and more often did sedentary work behind a computer. 34% were fully and 22% were partially medically retired

Note: Nivel and TNO are two independent Dutch research institutes.

**Table 4:** Cognitive behavioural therapy (CBT) and/or graded exercise therapy (GET) studies reporting on work status.

Study	Intervention	*n*	Criteria	Length of FU	Control Group	Work Outcome	Dropouts/Missing Data
Akagi et al. (2001) [108]	CBT; non-randomised noncontrolled study	94	Oxford or neurasthenia criteria, all labelled as ME/CFS	20 mo	No control group	Employment status increased from 15 to 27 patients. However, of those 27, 10 were on sick leave and 5 were unemployed. Also, 77% of those working changed occupation due to their illness	46% (43/94) dropped out
Bazelmans et al. (2005) [109]	Group CBT (GCBT), non-randomised trial	67 (patients with CFS or ICF)	Fukuda	6 mo	Waiting list (WL)	No statistically significant difference in hours worked per week at follow-up: 6.4 (GCBT) vs. 6.7 (WL; *p* = 0.958)	3% (2/67) dropped out from GCBT; 0% from WL
Burgess et al. (2012) [110]	Face-to-face CBT versus telephone CBT	80 (35 CBT, 45 telephone CBT)	Fulfilling both Fukuda and Oxford criteria	12 mo	No control group	Job to return to at baseline: 45.5% CBT and 21.9% telephone CBT. No employment data provided at follow-up.	34.3% (12/35 CBT) and 55.6% (25/45 telephone CBT) dropped out
Collin and Crawley (2017) [111]	Evaluation of CBT and GET in 11 NHS CFS clinics	952	NICE criteria	1 yr	No control group; evaluation of NHS treatment	After NHS treatment: 47.2% no change in employment situation; 18.0% returned to work or increased hours; 30.0% stopped working or reduced hours due to CFS and 4.8% for other reasons. 78.8% no change in education; 4.6% returned to or increased hours of education whilst 12.9% ceased or reduced these hours.	Response rate: 46.2% (440/952)
Cox (1999 and 2002) [112,113]	Inpatient Occupational Therapy Programme (IOTP) consisting of CBT and GET; non-randomised study	97 (61 inpatients + comparison group of 36 patients recruited from the pending inpatient admission list).	Fukuda	6 months post-discharge	No treatment control group (waiting-list)	At baseline not working: 92% IOTP, 97% WL; student 25% IOTP, 11% WL; housewife 0% IOTP, 3% WL; unemployed 5% IOTP, 8% WL. No employment data provided at follow-up.	Response rate: 70.5% (43/61) IOTP and 54% (19/36) WL
Deale et al. (2001) [114]	CBT	60	Oxford	5 yrs	Relaxation, poorly matched	No differences between groups in employment status at 5 year FU (p=0.28)	Dropouts: 16.7% (5/30) CBT, 6.7% (2/30) Relax
Dyck et al, (1996) [115]	Rehabilitation programme which included CBT and GET	2	Fukuda	3 mo	No control group	1 made a career change, the other one tried modified work	No drop outs
Friedberg et al. (2016) [116]	Fatigue self-management programme (CBT delivered by booklet and audio CDs) in severe CFS with web diaries and actigraphs; second group with less expensive paper diaries	137 patients with severe CFS	Fukuda	12 mo	Usual care control	At baseline 15.3% (21/137) worked full time, 21.2% (29/137) part-time or half time, 15.3% (21/137) were unemployed and 54.7% (75/137) disabled (participants were able to select multiple employment status categories). No employment data provided at follow-up. Actigraphy, step counter and six minute walk test showed no significant objective change.	5.1% (7/137) dropout rate
Fulcher and White (1997) [117]	GET	66	Oxford	12 mo	Flexibility exercises and relaxation therapy. Poorly matched groups; concerns if this was in fact a trial for ME/CFS patients	At baseline 39% (26/66) were working or studying at least part time, compared with 47% (31/66) after treatment	21% (14/66) dropped out
Hlavaty et al. (2011) [118]	CBT with graded activity, homework compliance	82 (divided over 4 treatment groups)	Fukuda	12 mo	3 other treatment groups: cognitive coping skills, relaxation or anaerobic exercises	At baseline 57.3% were retired, unemployed or on disability; 37.9% worked full-time or part-time; 1.2% working and on disability. No employment data provided at follow-up.	Unclear
Huibers et al. (2004) [119]	CBT delivered by GPs	151 fatigued employees on sick leave (66 met CFS criteria)	Fukuda	12 mo	No treatment	At 4 mo 50% (CBT) and 61% (NT) and at 12 mo 59% (CBT) and 65% (NT) resumed work	Did not complete: 33% (25/76) CBT, 9.3% (7/75) no treatment
Janse et al. (2017) [120]	Evaluation of four studies: 2 of CBT, 1 of group CBT and 1 of stepped care CBT *	583 (participants from four trials grouped together)	Fukuda	5 yrs, minimum of 18 mo	No control groups (2 non-randomised noncontrolled studies, one randomised study had no control group and control group from 4th study was not used for this evaluation)	At long-term FU, 54% (264/490) had paid work and 27% (114/430) received a disability pension. Baseline employment data was not provided.	Response rate was 84.0% (490/583, paid work) and 73.8% (430/583, disability pension) respectively. The authors themselves noted that non-responders scored significantly lower on physical functioning at short-term follow-up than responders.
Janse et al. (2018) [121]	Protocol iCBT vs. on demand iCBT **	240	Fukuda	6 mo	Waiting-list (WL)	Paid job at baseline: 65% Protocol iCBT, 71% on demand iCBT and 68% WL. No employment data provided at follow-up.	Dropped out: 6.3% (5/80) Protocol iCBT, 8.8% (7/80) on demand iCBT, 5% (4/80) WL
Jason et al. (2007) [122]	CBT vs. cognitive therapy vs. anaerobic activity	114	Fukuda	12 mo	Relaxation	At baseline, 19.3% were working full time, 20.2% part time, 24.6% on disability, 23.7% unemployed, 6.1% retired, 4.4% part-time students, 0.9% full time students and 0.9% working part time and on disability. No significant interaction effects were found for employment at FU.	25% dropped out; no differences between groups
Koolhaas et al. (2009) [123]	CBT; patient survey by University	100	98% diagnosed by a doctor, 2% by a psychologist	Patient survey	No control group	41% worked before, 31% after CBT; patients who were able to work, worked five hours per week less after CBT	Not applicable
Lopez et al. (2011) [124]	Cognitive behavioral stress management (CBSM)	69 (44 CBSM, 25 control group)	Fukuda	12 weeks (end of treatment)	Psycho educational (PE) seminar	At baseline: 13.2% worked full time, 18.8% part time, 15.9% unemployed 4.3% retired, 2.9% student and 44.9% on disability. No employment data provided at follow-up.	13.6% (6/44) CBSM and 20% (5/25) PE lost to FU
Marlin et al. (1998) [125]	Multidisciplinary intervention (MDI) that include CBT; 50% also treated with full dose antidepressants	71 (51 MDI, 20 control) nonrandomised study with patients from a private clinic	Fukuda	33 mo	No treatment control group (many of them had declined MDI)	Average duration of work disability at baseline: 23 mo MDI, 39 mo NT; at FU: 25 mo MDI, 27 mo NT.	69% (49/71) were lost to follow up.
Masuda et al. (2002) [126]	Multidisciplinary treatment *** for both treatment groups	38 (9 postinfectious (PI) and 9 non-infectious (NI) CFS; 20 HC) non-randomised study	CDC 1988	2 yr	No treatment	Illness duration: 8.2 mo PI and 38.2 mo NI (badly matched). Postinfectious group: 3 returned to work, 5 others changed occupation or workplace; non-infectious group: 3 returned to work. No employment date for HC.	No drop outs
McBride et al. (2017) [127]	Online cognitive remediation training programme including CBT+GET (OCRTP) **** vs. CBT+GET alone (CGA). Non-randomised trial	72 (36 in each group)	Fukuda	12 wks	No control group	Baseline characteristics: currently employed 33% CGA, 22% OCRTP; disability pension 14% CGA, 8% OCRTP; hours of employment/week 6 CGA, 19 OCRTP; currently studying 27% CGA, 25% OCRTP; hours of study/week 6 CGA, 11 OCRTP. No employment data provided at follow-up.	Unclear
McDermott et al. (2004) [128]	Lifestyle management programme based on CBT+GET with pacing as the core strategy	98, nonrandomised trial	Fukuda	18 mo	No control group	Of those who attended 4 or more sessions of therapy, 8.5% (5/59) returned to work full time and 10.2% (6/59) part-time	24.5% (24/98) lost to follow up; 79.7% (59/74) completed at least four sessions of treatment
Moss-Morris et al. (2005) [129]	GET	49 self referred patients from a CFS private practice (25 GET, 24 controls)	Fukuda	6 mo	No treatment, poorly matched control group	22.4% were unemployed and unable to work due to disability at baseline, No employment data provided at follow-up. Fitness (VO2peak) deteriorated by 15% after GET	Lost to FU: 36% (9/25) GET, 29.2% (7/24) no treatment
O'Dowd et al. (2006) [130]	Group CBT incorporating graded activity vs. education and support group	153	Fukuda	12 mo	No treatment (SMC)	The authors concluded that group CBT did not significantly improve employment status.	Missing cognitive test data: 28.9% CBT, 13.7% NT
Prins et al. (2001) [131]	CBT versus guided support	278	Oxford	14 mo	No treatment (natural course).	No statistically significant difference in number of hours worked at 8 (*p* = 0.3362) and 14 mo (*p* = 0.1134) between CBT and natural course	40.9% (55/93) CBT and 23.1% (70/91) no treatment (dropouts)
Powell et al. (2001) and (2004) [132,133]	GET vs. telephone intervention with GET vs. minimum intervention with GET	148	Oxford	2 yrs	No treatment (NT; labelled as SMC; participants received an information booklet that encouraged graded activity and positive thinking)	At baseline: working: 39.5% (15/38) GET, 35.1% (13/37) minimum, 28.2% (11/39) telephone, 32.4% (11/34) NT. Disability benefit: 42.1% (16/38) GET, 17/37 minimum, 16/39 telephone, 15/34 NT. No employment data provided at follow-up.	Response rate: 77.0% (114/148)
Ridsdale et al. (2001) [134]	CBT versus counseling	160 (fatigue study, 28% (45) had CFS)	Fukuda	6 mo	Counselling	At baseline 3.1% (counselling) and 10.9% (CBT) were off sick. Days off work improved by 4.3% (15/350, counselling) vs. deteriorated by 6.6% (55/829, CBT) [135]	36% (29/80) counselling and 31% (25/80) CBT dropped out
Ridsdale et al. (2004) [136]	CBT vs. GET	123 (fatigue study, 29% (36 patients) fulfilled Fukuda criteria (*n* = 15 CBT, *n* = 21 GET)	Fukuda	8 mo	Post hoc added non-randomised prospective no treatment control group; badly matched. Patients were given a booklet on self-management of fatigue	Employed at base line: 60% CBT, 73% GET vs. 65% control group. No employment data provided at follow-up. Step test results not published.	29% (18/63) CBT and 40% (24/60) GET did not complete 6 sessions of therapy; 22.5% (9/40) did not provide follow-up data (control group)
Sandler et al. (2016) [137]	Integrated programme of CBT, GET and pacing. Non-randomised noncontrolled trial	264 (245 CFS and 19 post-cancer fatigue (PCF) patients)	Fukuda (for CFS)	24 weeks	No control group	At baseline 39% (104/264) in receipt of sickness benefits or medical pension. Unclear how many of those had CFS. No other employment data provided. Also, no employment data provided at follow-up.	36% (96/264) missing data
Saxty et al. (2005) [138]	Group CBT, non-randomised nonnoncontrolled trial	6	Fukuda	3 mo	No control group	At baseline 1 was working full time, 3 part-time and 2 were on sick leave. At follow-up, the 2 part-time workers had increased their hours. No other employment changes	No drop outs; therapy attendance rate 86.7%
Scheeres et al. (2008) [139]	CBT; non-randomised noncontrolled study	125 (13 did not fulfil the Fukuda criteria)	Fukuda	8 mo	No control group	At baseline 62% had a paid job. Fewer patients had a paid job after treatment than before (percentage not give). The number of contract hours after CBT decreased from 16.2 to 14.9 but the number of hours worked increased from 9.4 to 11.4 per week.	35.7% (40/112) dropped out; the last observation was used in case of missing data
Schreurs et al. (2011) [140]	CBT combined with GET (inpatient rehabilitation programme); non-randomised noncontrolled study	160	Fukuda	6 mo	No control group	At intake 52% (83/160) on disability benefits, 31.2% (50/160) were working mostly part-time, 2.5% (4/160) had own business, 8.8% (14/160) were school going. No employment data provided at follow-up.	27% (44/160) no FU measurements
Stordeur et al. (2008) [141]	Evaluation of CBT and GET in Belgium CFS knowledge centres	655	Fukuda	Treatment evaluation	No control group (treatment evaluation)	Employment status decreased from 18.3% to 14.9%; sickness allowance status increased from 54% to 57%	28% dropped out
Vos-Vromans et al. (2016 and 2017) [142,143]	Multidisciplinary rehabilitation treatment (MRT) which contained an element of CBT versus CBT	122	Fukuda	12 mo	No control group	At baseline 68% (39/57) MRT and 52% (27/52) CBT had paid work and were working 26.1 (MRT) and 29.8 (CBT) hours per week. No employment data provided at follow-up. Objective improvement: 5.8% MRT and 6.5% CBT (activity monitor)	20% (12/60) CBT and 10% (6/62) MRT (dropped out)
Wearden et al. (1998) [144]	2 treatment groups: exercise and 20 mg fluoxetine versus appointments only and 20 mg fluoxetine	136	Oxford	6 mo	2 control groups: exercise and placebo drug; appointments and placebo drug	At baseline 84% had changed occupation. No employment data provided at follow-up.	37% (25/67, exercise) vs. 22% (15/69, non-exercise) (dropouts); drop-outs were significantly more likely than trial completers to have changed or given up their occupation as a result of their illness (95% vs. 79%); 34% (23/67) complied fully with GET, 78% (54/69) complied fully with exercise placebo
Wearden · et al. (2010, 2012 and 2013) [145,146,147]	Pragmatic rehabilitation (CBT, GET and explanation about CFS to patients) vs. supportive listening	296	Oxford	70 wks	No treatment (GP treatment as usual)	At baseline 65% (187/296) in receipt of benefits. No other employment data provided at baseline or follow-up. Step test showed no objective improvement	13% (39/296) dropped out
White et al. (2011) [148]	CBT vs. GET vs. APT (all 3 also contained SMC)	641	Oxford	52 wks (with long-term follow-up (LTFU) at 31 months [149]).	SMC (no treatment)	Lost employment: remained 84% (CBT); increased from 83% at baseline to 86% (GET) at FU. Income benefits increased from 10% to 13% (CBT) and from 14% to 20% (GET); illness/disability benefits increased from 32% to 38% (CBT) and from 31% to 36% (GET); payments from income protection schemes or private pensions increased from 6% to 12% (CBT) and from 8% to 16% (GET) [150]. No employment data provided at LTFU.	Dropouts: 10.5% (17/161) CBT, 6.3% (10/160) GET. Missing step test data: 33.8% (54/160) GET and 29.8% (48/161) CBT [151]
Wittkowski et al. (2004) [152]	Group CBT; non- randomised noncontrolled trial	6	Fukuda	3 mo	No control group	1 returned to full-time employment and 1 worked part-time on a phased return	33% (2/6) dropped out
Worm-Smeitink et al. (2016) [153]	Comparison of efficacy of CBT in two leading international centres (UK and Netherlands)	NL: 293, UK: 163	NL: Fukuda, UK: Oxford	Unclear	Outcomes after CBT in the other country	At baseline employed: 67.6% NL, 55.2% UK; number of hours worked: 9.88 NL, 13.80 UK; on sick leave: 51.5% NL, 20% UK. No employment data provided at follow-up.	Dropped out: NL: 7.8% (23/293), UK: 6.7% (11/163)
Worm-Smeitink et al. (2019) [154]	Prescheduled or on-demand internet-based CBT (iCBT) followed by face-to-face (f2f) CBT when necessary versus f2f CBT *****	363	Fukuda (7 patients had <4 of the required 4 or more additional symptoms)	Unclear	No control group	Paid job at baseline: 68.9% prescheduled iCBT, 65.8% on-demand iCBT, 64.7% f2f CBT. No employment data provided at follow-up.	Dropped out plus <4 CDC criteria: 5% (6/121) prescheduled iCBT, 11.6% (14/121) on-demand iCBT, 31.4% (38/121) f2f CBT; of those who met step-up criteria 55.4% (51/92) prescheduled iCBT and 41.9% (39/93) on-demand iCBT declined f2f CBT

APT: adaptive pacing therapy; FU: follow-up; HC: healthy controls; ICF: idiopathic chronic fatigue; NT: no treatment; SMC: specialist medical care; vs.: versus; WL: waiting list. * Janse et al. (2017) [120] reviewed the efficacy of different forms of CBT of the following four trials at long-term follow-up (5 years with a minimum of 18 months). Heins et al. [155] (*n* = 232) and Knoop et al. [156] (*n* = 112) were 2 non-randomised noncontrolled trials of CBT. Tummers et al. [157] (*n* = 171) was a randomised trial without a control group. They compared guided self-instruction, followed by CBT with CBT alone. Wiborg et al. [158] (*n* = 204) was a waiting-list controlled trial of group CBT. This wait-list group (*n* = 68) was not assessed by Janse et al. Consequently, all 4 studies in the review by Janse et al. (2017) were noncontrolled. ** Janse et al. (2018) [121]: Protocol iCBT: Internet-based CBT with protocol-driven therapist feedback; on demand iCBT: Internet-based CBT with therapist feedback on demand. *** Masuda et al. (2002) [126]: Multidisciplinary treatment consisting of three-stage treatment programs was carried out for all patients. Each stage of treatment required 3 weeks. The 1st stage consisted of drug therapy, rehabilitation, and counseling. The second stage consisted of cognitive behavioral therapy and family therapy, and the third stage consisted of exercise therapy. **** McBride et al. (2017) [127]: online cognitive remediation training programme (OCRTP; cognitive exercise therapy; CET) in addition to CBT+GET compared to CBT+GET alone (CGA). ***** Worm-Smeitink et al. (2019) [154] was a three-arm, parallel, randomized, noninferiority trial. In 2 arms, the patients received stepped care (SC) consisting of I-CBT, either with protocol-driven feedback (SC-protocol-driven feedback) or with feedback on demand (SC-feedback-on-demand), followed by face-to-face (f2f) CBT when necessary, that is, still severely fatigued (CIS fatigue severity >35) or disabled (SIP >700) after I-CBT. The third arm was f2f CBT after a variable waiting period.
